# Comparison of Activation in the Prefrontal Cortex of Native Speakers of Mandarin by Ability of Japanese as a Second Language Using a Novel Speaking Task

**DOI:** 10.3390/healthcare9040412

**Published:** 2021-04-02

**Authors:** Li Cong, Hideki Miyaguchi, Chinami Ishizuki

**Affiliations:** 1 Graduate School of Biomedical & Health Sciences, Hiroshima University, Hiroshima City 734-8551, Japan; l-congli@hirokoku-u.ac.jp; 2 Faculty of Rehabilitation, Hiroshima International University, Higashi-Hiroshima City 739-2695, Japan; 3 Department of Human Behavior Science of Occupational Therapy, Health Sciences Major, Graduate School of Biomedical and Health Sciences, Hiroshima University, Hiroshima City 734-8551, Japan; ishizuki@hiroshima-u.ac.jp

**Keywords:** bilingualism, Mandarin/Japanese, functional brain imaging, prefrontal cortex, speaking task, functional near-infrared spectroscopy, cognitive load, inhibition

## Abstract

Evidence shows that second language (L2) learning affects cognitive function. Here in this work, we compared brain activation in native speakers of Mandarin (L1) who speak Japanese (L2) between and within two groups (high and low L2 ability) to determine the effect of L2 ability in L1 and L2 speaking tasks, and to map brain regions involved in both tasks. The brain activation during task performance was determined using prefrontal cortex blood flow as a proxy, measured by functional near-infrared spectroscopy (fNIRS). People with low L2 ability showed much more brain activation when speaking L2 than when speaking L1. People with high L2 ability showed high-level brain activation when speaking either L2 or L1. Almost the same high-level brain activation was observed in both ability groups when speaking L2. The high level of activation in people with high L2 ability when speaking either L2 or L1 suggested strong inhibition of the non-spoken language. A wider area of brain activation in people with low compared with high L2 ability when speaking L2 is considered to be attributed to the cognitive load involved in code-switching L1 to L2 with strong inhibition of L1 and the cognitive load involved in using L2.

## 1. Introduction

Humans learn their first language (hereinafter referred to as L1) naturally from their parents in parallel with lateralization of the brain. A mostly right-handed person has their language center in the left hemisphere. Both the Wernicke and the Broca areas in the left hemisphere become active when people are trying to understand or express something in language [1]. Antoniou et al. [2] elucidated how the prefrontal cortex was involved in learning a second language (hereinafter referred to as L2). Rodriguez-Fornells et al. [3] reported that the prefrontal cortex, especially Brodmann Areas (BA10 and BA46), is particularly involved in the early stages of L2 acquisition. Additionally, it was reported that the volume of white matter in the prefrontal cortex of the right hemisphere increases and neural bonds strengthen with L2 acquisition [4]. Moreover, density of both gray matter and white matter was revealed to have increased with L2 acquisition [5,6,7] Furthermore, patterns of brain activation were associated with age of L2 learning, task difficulty, and proficiency of L2 ability.

Onset of dementia in bilinguals is about 4–6 years later than in monolinguals, according to a large-scale investigation [8]. The reason is believed to lie in cognitive processes involved in inhibition of one language in favor of another while code-switching between languages. It was suggested that use of multiple languages over many years requiring code-switching and inhibition affects cognitive function [6,9,10]. Additionally, it has been reported that the anterior middle frontal gyrus, especially BA46, plays a central role in language production and is involved in control of cognitive function [11]. Evidence that the prefrontal cortex is involved in switching between languages was demonstrated by brain activation in this location during code-switching tasks [12].

In tasks requiring repetitive code-switching, it was confirmed that the number of times required for code-switching was higher in bilinguals than in less proficient speakers of the second language, and reaction time was shorter in the former [9,13]. Accordingly, it has been well documented that there are structural, functional, and cognitive associations between language function and the prefrontal cortex activity of bilinguals. Therefore, it was suggested that L2 learning affects cognitive function [14,15]. Nowadays, with increasing longevity worldwide, and considering the onset of dementia is delayed in second-language speakers, this is an important area of research.

Regarding the relationship between brain activation and language proficiency, there have been many studies of bilinguals including Japanese/English (L1/L2) [4] and English/Mandarin (L1/L2) speakers [5]. Among these languages, English uses phonetic characters, whereas Mandarin and Japanese use ideographs. It can be expected that brain activation patterns may differ slightly due to the differences among these languages, and that brain activity patterns may change when the code-switching function is activated or repeatedly employed. In particular, Mandarin and Japanese use almost the same *Kanji* characters (漢字), but their pronunciation and grammar differ. In Mandarin, the order of *Kanji* characters in speech or text is closer to that of English as subject–verb–object (SVO), but that of Japanese is (S)OV. Additionally, Mandarin contains only *Kanji*, whereas Japanese includes *Hiragana* and *Katakana* too. *Katakana* was derived from English, Latin, and other languages. There are many differences between Mandarin and Japanese languages due to their different historical backgrounds and cultures. It would be useful to clarify how L2 proficiency affects prefrontal cortex activation in people speaking Mandarin/Japanese (L1/L2). As far as the authors are aware, no previous study has investigated this.

Here, a novel speaking task was developed wherein Mandarin/Japanese (L1/L2) speakers had to describe stimuli using L1 or L2. Simultaneously, cerebral blood flow changes in the prefrontal cortex as proxy for prefrontal cortex activation were analyzed. The relationships between such activation and L2 proficiency were analyzed and discussed.

## 2. Methods

### 2.1. Subjects

Twenty-four right-handed, healthy Chinese speakers of Mandarin with Japanese as a second language were divided into low- and high-L2-ability groups, determined by self-evaluation questionnaire with both L1 and L2 scored on a scale of 1 to 10 in all four domains: listening, speaking, writing, and reading [16,17,18]. Each individual’s self-evaluation was obtained according to the guideline of 1 (very poor level), 5 (adequate level), or 10 (perfect level). Those with high L2 ability had lived in Japan for over 20 years as adults and used Japanese in their daily activities and Mandarin at home. They were essentially bilingual. In contrast, those with low L2 ability were graduate students who had lived in Japan for only two years or so. Although they spoke Japanese, their Mandarin proficiency was clearly higher. In addition, cognitive reserve was measured using Cognitive Reserve Index questionnaire (CRiq) [19].

Characteristics of the study participants with standard deviation (SD) and *p*-value are shown in Table 1. Ethical approval for the present study was obtained from Hiroshima International University, and the study adhered to the protocols of the Helsinki Declaration. All subjects provided written informed consent.

### 2.2. Speaking Task

**Figure 1:**
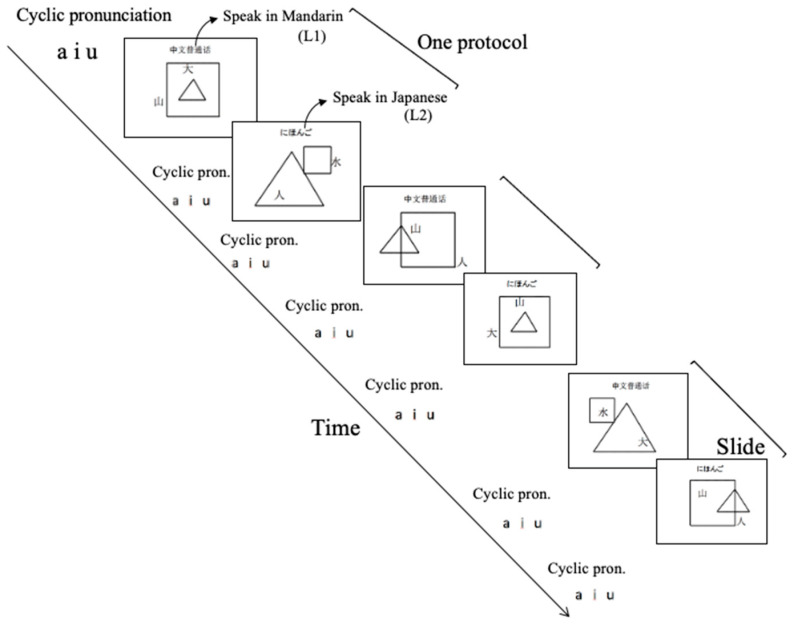
Speaking task slide schedule.

Subjects were tasked to describe in Japanese or Mandarin stimuli that appeared on a PC screen in the sequence of 15 s pre-rest–30 s speaking task–15 s post-rest, as shown in Figure 1. Briefly, six PowerPoint slides displaying monochrome kanji characters, shared by both languages but with different pronunciations of mountain (*山*—shan/yama), large (*大*—da/dai, people (*人*, ren/hito), and water (*水*, shui/mizu), and shapes (triangle *△*—sanjiao/sankaku, (rectangle *□*—sijiao/shikaku) of different sizes and locations were presented, and subjects were tasked to describe the stimuli using either L1 or L2. After subjects confirmed that they understood the task requirements, the experimenter retreated out of vision of the subjects and the slide show commenced. The target language was indicated at the top of each slide. Between stimuli slides, a slide instructing subjects to repeatedly pronounce at normal conversation speed the vowels “a” (*阿* in Mandarin or *あ* in Japanese), “i” (*伊* or *い*), and “u” (*烏* or *う*) in order for 30 s was presented, which was deemed to represent 15 s pre- and post-rest periods and was used to obtain baseline. The slide show progressed regardless of whether or not subjects had completed their responses to stimuli slides.

### 2.3. Measurement Environment

**Figure 2:**
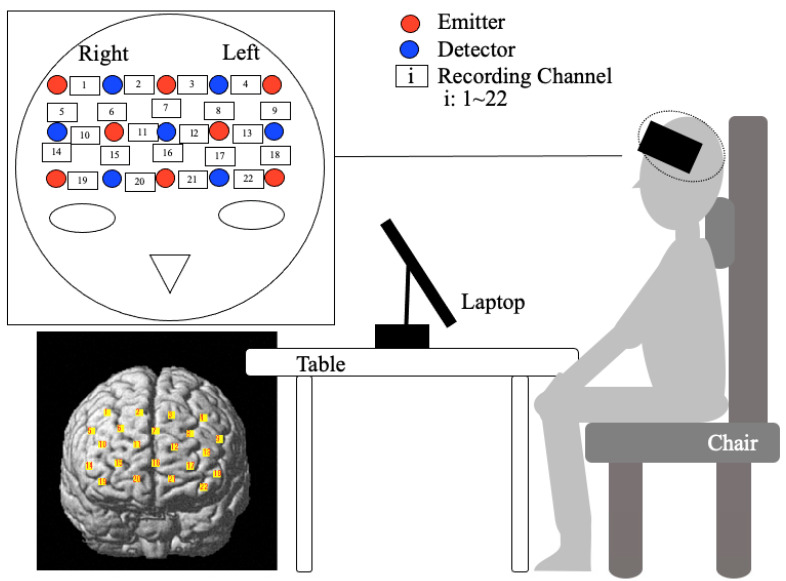
Arrangement of sensor array and 22 channels above the prefrontal cortex.

The tasks were performed in a quiet room under adequate lighting with the temperature maintained at about 25 °C. The subjects sat on a seat in an upright position and were instructed to maintain a still posture with their hands on their knees and to keep their head still, which was supported by a cushion as shown in Figure 2, while wearing a device to record and measure brain activation.

### 2.4. Measuring Positions

Data of localized blood oxygenation levels in the prefrontal cortex indicating neural activity were acquired by a functional near-infrared spectroscopy (fNIRS) system that included an array of sensors (FOIRE-3000, Shimadzu Co. Japan) worn on the head, which recorded change in cerebral blood flow during task performance. The array of sensors (fNIRS sources and detectors) was equipped with 22 channels and was attached to the head in a location positioned from the prefrontal area in accordance with the International 10–20 system (Figure 2). The sensors were positioned across from each other at 3 cm intervals. Basing on the modified Beer–Lambert law, the oxy-hemoglobin change (Δoxy-Hb, mM·mm) was acquired from the cortical concentration levels. The sites to measure oxy-hemoglobin change associated with cerebral blood flow change were determined using a 3D digitizer (FASTRACK, Polhemus) as previously described [20,21]. Their placements coincided with Brodmann Areas BA9, BA10, and BA46. The physiological noise from cardiac signal and respiration, and so forth was filtered by a temporal low-pass cut-off at 0.1 Hz.

### 2.5. Data Analysis

#### 2.5.1. Approximate Integrals of Cerebral Blood Flow Change

**Figure 3:**
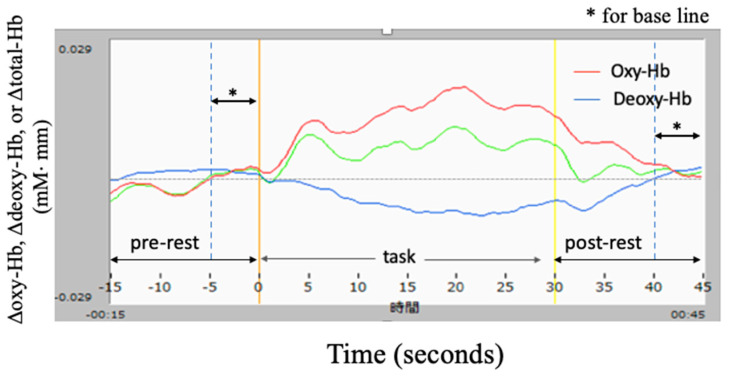
Sample waveforms of cerebral blood flow obtained from a channel in a subject: red, Δoxy-Hb (oxy-hemoglobin change); blue, Δdeoxy-Hb; green, Δtotal-Hb.

Figure 3 shows sample waveforms of cerebral blood flow change obtained from a channel in a subject. Red, blue, and green lines show change in Δoxy-Hb, Δdeoxy-Hb, and Δtotal-Hb, respectively. Each line was smoothed by 5 data (sampling rate: 0.13 s/datum) for three times. The data obtained during the 5 s pre- and post-rest period were taken as baseline data for comparison within subjects.

The data of Δoxy-Hb obtained during performance of the speaking task were approximately integrated for analysis of cerebral blood flow change using a method previously described [22]. Note that Δdeoxy-Hb was not used in the following analysis.

Since the data was parametric and showed a normal distribution, comparisons between groups were assessed using Student’s *t*-test with differences with a probability of *p* < 0.05 deemed significant. Additionally, the correlations between the L2 ability and the cerebral blood flow changes while speaking each language were obtained by linear regression analysis using the least-squares estimation.

#### 2.5.2. Common Activation Regions

To map brain regions that were commonly activated during task performance, the data of Δoxy-Hb were treated using a Statistical Parametric Mapping software package (NIRS-SPM; Welcome Trust Centre for Neuroimaging, London, UK) run in a MATLAB-based environment. This treatment is frequently applied when dealing with magnetic resonance imaging by using the general linear model analysis as described [23], after excluding the activations caused by non-task factors such as subjects’ body movement. The temporal autocorrelation was estimated and then removed through a Gaussian smoothing with a full width at half maximum at two seconds. A detrending algorithm, which is based on the wavelet minimum description length, was applied to correct the signal distortion. The beta value as the individual task-related activity was obtained from a general linear model analysis with the hemodynamic response curve to model the Δoxy-Hb values. The topography was drawn from the beta values, which correspond to the sites of sensors. When the SPM t-statistic maps were calculated for group analysis, the common regions of activation were determined as significantly (*p* < 0.05) more active than others during the task performance.

## 3. Results

### 3.1. Language Proficiency

**Figure 4:**
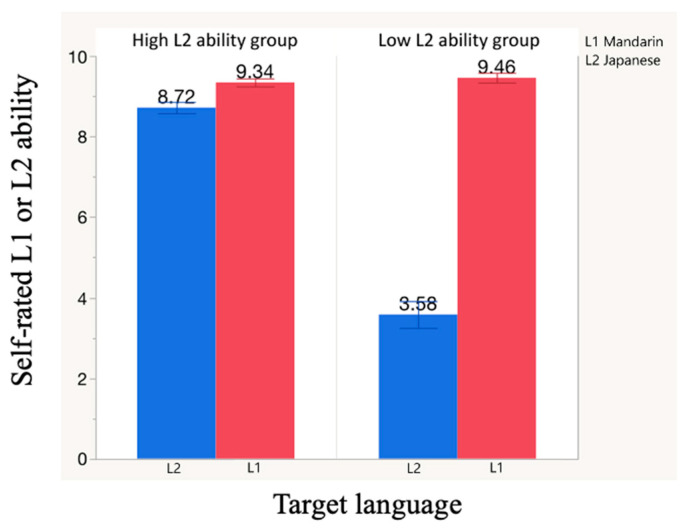
Self-rated L1 and L2 ability of native speakers of Mandarin (L1) who speak Japanese (L2).

Results of the self-assessed language proficiency questionnaire are presented in Table 1. Figure 4 shows the mean scores with standard deviation for L1 and L2 overall ability in both L2-ability groups. In the high-L2-ability group, there was no significant difference between L1 and L2 ability. In the low-L2-ability group, L2 ability was significantly lower than L1 ability (*p* < 0.001). There was no significant between-group difference in L1 ability.

### 3.2. Cerebral Blood Flow Change in the Prefrontal Cortex

**Figure 5:**
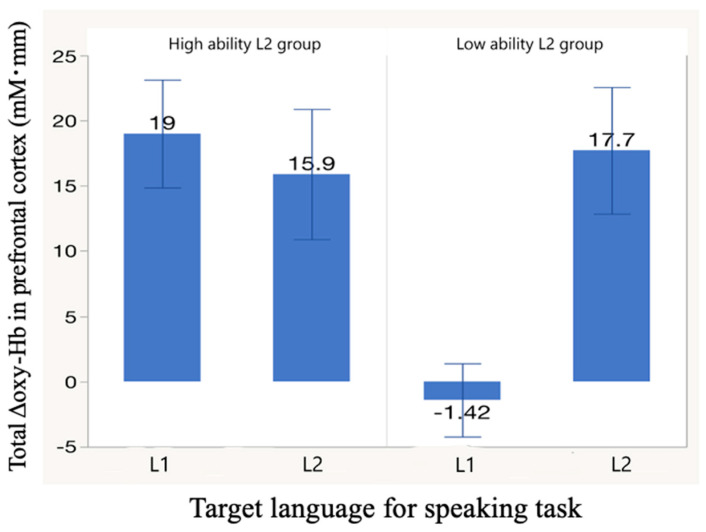
Approximate integral values of blood flow change (Δoxy-Hb) in the prefrontal cortex during speaking tasks.

Figure 5 shows approximate integral values of Δoxy-Hb observed in the prefrontal cortex for both high- and low-L2-ability speakers in performance of a speaking task. The horizontal axis indicates the speaking task target language. The vertical axis indicates the integrated amounts of Δoxy-Hb measured in the prefrontal cortex. Error bars indicate the standard deviation.

In high-L2-ability speakers, the value of Δoxy-Hb in the prefrontal cortex was slightly higher when speaking L1 than when speaking L2, albeit not significantly. In contrast, in low-L2-ability speakers, the value was significantly lower when speaking L1 than when speaking L2. Moreover, the value of Δoxy-Hb in the high-L2-ability speakers was significantly higher than that in the low-L2-ability speakers when speaking L1 (*p* < 0.005). Furthermore, there was no significant between-group difference when speaking L2 (*p* = 0.795).

### 3.3. Cerebral Blood Flow Change in the Left and Right Prefrontal Cortices

**Figure 6:**
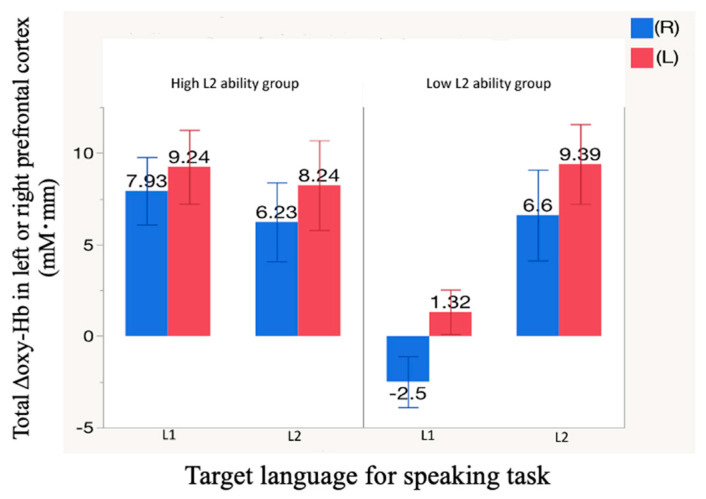
Values of Δoxy-Hb in the left and right prefrontal cortex of native speakers of Mandarin by L2 ability.

Figure 6 compares Δoxy-Hb values with standard deviation in left and right hemispheres of the prefrontal cortex within groups for both speaking tasks. In high-L2-ability speakers, the values were higher in the left than in the right hemisphere whichever language was spoken. The same was true in low-L2-ability speakers, however in these subjects the value in the right hemisphere was below the baseline value during performance of the L1 task.

### 3.4. Cerebral Blood Flow Change at Each of the 22 Channels

Table 2 compares mean ± SD of values of cerebral blood flow change and significance differences between high- and low-L2-ability speakers at each channel during performance of the speaking tasks. High-L2-ability speakers showed significantly higher values than those of low-L2-ability speakers, in all channels but 3, 4, and 8 located in the left dorsolateral prefrontal cortex when speaking L1 (*p* < 0.05). In contrast, there was no between-group difference in any channel when speaking L2.

Table 3 compares mean ± SD of values of cerebral blood flow change between speaking tasks in high-L2-ability speakers at each channel. No significant difference was observed at any channel.

Table 4 compares mean ± SD of values of cerebral blood flow change between speaking tasks in low-L2-ability speakers at each channel. When speaking L1, values at most channels in the right frontal cortex of these speakers tended to be below the baseline value. Consequently, when speaking L2, the values at those channels were significantly higher (*p* < 0.05).

### 3.5. Correlations between Cerebral Blood Flow Change and Language Proficiency

**Figure 7:**
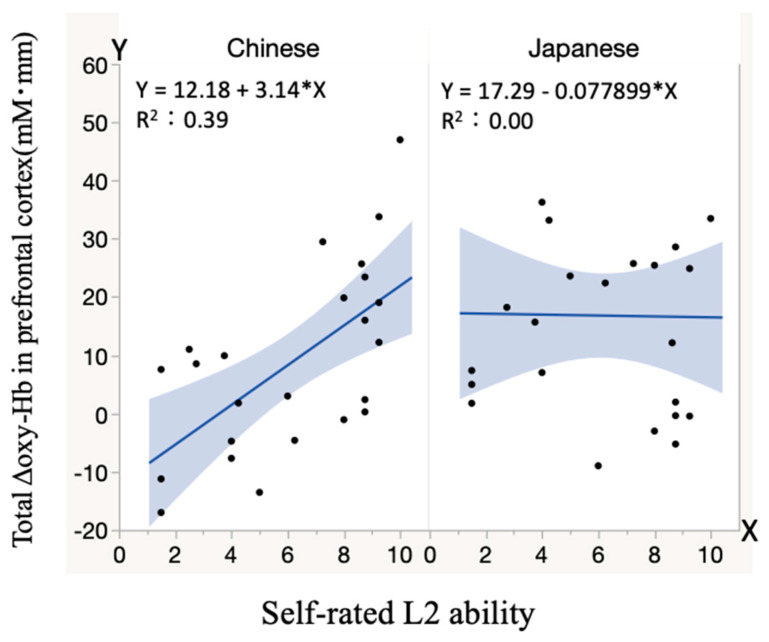
Correlations between L2 ability of all subjects and cerebral blood flow change during speaking either language.

Figure 7 shows relations between L2 ability and values of cerebral blood flow change in the prefrontal cortex when speaking each language. The solid lines were obtained by linear regression analysis using the least-squares estimation. With increase in L2 ability, cerebral blood flow increased when speaking L1. A correlation coefficient (*R*) of 0.62 corresponding to a coefficient of determination (*R*^2^) of 0.39 was obtained, indicating strong correlation between L2 ability and values of Δoxy-Hb with clear predictability. On the other hand, there was no correlation between L2 ability and blood flow change when speaking L2.

### 3.6. Common Activation Area Obtained from NIRS-SPM Analysis

**Figure 8:**
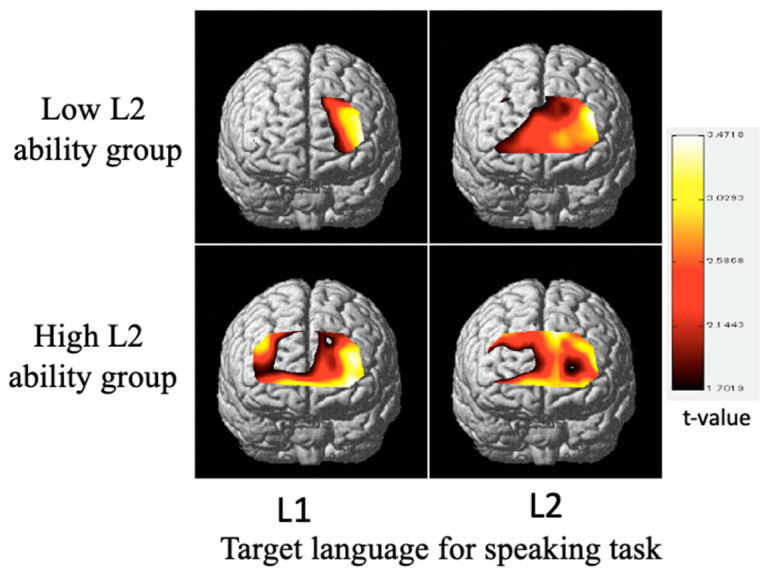
Regions of common activation, obtained by NIRS-SPM, in the prefrontal cortex of native speakers of Mandarin (L1) who speak Japanese (L2) by L2 ability (*p* < 0.05).

Figure 8 depicts common activation regions in both low- and high-L2-ability speakers speaking L1 and L2, which were determined as those areas significantly (*p* < 0.05) more activated than other areas during task performance. In low-L2-ability speakers, regions BA9 and BA46 in the left dorsolateral prefrontal cortex (DLPFC), corresponding to channels 3, 4, 8, and 9, were commonly activated when speaking L1. And regions BA9 and BA46 in the DLPFC, and BA10 in the frontal pole, corresponding to channels 3, 4, 7–9, 12, 13, and 16–22, were commonly activated when they spoke L2. In high-L2-ability speakers, not only the left but also the right DLPFC was activated when speaking L1, and regions in both the right and left hemispheres were activated when speaking L2.

## 4. Discussion

In low-L2-ability speakers, activation was detected in the left side of the brain only when speaking L1, but when speaking L2 their activation region expanded to a wide range in the frontal cortex, including the frontal pole. In contrast, in high-L2-ability speakers, both sides of the brain were activated in either task. It is suggested that the activation pattern of the prefrontal cortex changes with language learning experience and proficiency, and thus the cortex and gray matter were physically influenced. The above results give new evidence that the experience of L2 learning affects prefrontal cortex function.

### 4.1. Subjects Selection and Cognitive Reserve Unification

To perform the speaking tasks in this study, some minimum proficiency in Japanese was necessary. Early Japanese learners might be nervous and use hand or body gestures in the Japanese task, which had been confirmed by a pilot experiment. This might cause significant bias in frontal lobe measurements. Therefore, graduate students who had lived in Japan for two years or so and rated themselves as not high ability in the self-assessment questionnaire were defined as low-L2-ability speakers in this study, rather than including early Japanese leaners who spoke hardly any Japanese [24]. People who had lived in Japan for over 20 years who showed a slightly higher proficiency in Japanese than Mandarin, perhaps due to the frequency of occasions required to speak Japanese in their daily life, were defined as high-L2-ability speakers. In fact, during the L1 task, unlike the other group of subjects, some of these sometimes made the error of responding in L2. Activation measurements on those occasions were excluded from analysis.

To compare prefrontal cortex function between groups of subjects, it is essential that within the group members have similar prefrontal cortex function. In the present study, cognitive reserve in both groups was measured using Cognitive Reserve Index questionnaire (CRiq) [19], and scores of index 92 ± 3 and 120 ± 8 were obtained from low- and high-L2-ability groups, respectively. In this way, the cognitive reserve within each group was unified.

Furthermore, since age of learning a second language is strongly associated with physical change in gray matter and white matter pathways involved in language processing [25,26], we excluded young subjects and included only subjects who started to learn Japanese after reaching adulthood. All subjects were aged over 22 and learned Japanese after they came to Japan.

### 4.2. Validity of Experimental Conditions and Analysis Methods

To ensure intrasubject reproducibility of prefrontal cortex activation, the experiment procedure followed the protocol of the verbal fluency task [27], which is commonly used in Japan. Baseline values were obtained from repeated pronunciation of vowels common to both Mandarin and Japanese, (*阿*, Mandarin or *あ*, Japanese), (*伊* or *い*), and (*烏* or *う*), which are transcribed similarly as “a”, “i”, and “u” in roman characters. During the rest-task of repeated pronunciation of a, i, and u at normal conversation speed, cerebral blood flow change was confirmed to have low values, indicating the baseline task did not exert the subjects.

The differences between baseline activation values and activation levels observed during performance of the speaking tasks were assumed to be measures of cognitive language processing behavior. The baseline activation values themselves were assumed to be measures of physical language production behavior. Three protocols, as shown in Figure 1, were performed while cerebral blood flow change was observed and analyzed, as in a previous study [22]. The whole procedure took less than 10 min, including fitting the sensor array on the subject’s head.

### 4.3. Comparison of Brain Activation

Region BA9 in the right DLPFC (ch1, 2, 5) was activated significantly more in high than in low-L2-ability speakers when speaking L1. It is proposed that this reflected the demand of cognitive load to inhibit L2. In other words, significant cognitive load occurred when code-switching from Japanese to Mandarin. Note that code-switching was defined from various perspectives. Here in this work, it should be limited to sociolinguistics concerning bilinguals, which helps discuss the brain activation during task performance. Similar levels of cognitive load when speaking L2 likely occurred to inhibit L1. We suggest these high-L2-ability speakers had little difference in proficiency of either language, i.e., neither was dominant (equally bilingual), thus, to speak one language cognitive load was required to inhibit the other. This notion has been discussed previously ^22^.

In the low-L2-ability speakers, brain activation was similarly high and appeared over a fairly wide area when speaking L2 (Figure 5 and Figure 8). This could be attributed to (i) cognitive load demand in code-switching and inhibiting L1 and (ii) cognitive load demand in using L2. When speaking L1, either the cognitive load demand in inhibiting L2 (i) or that in using L1 (ii) was lower. Therefore, low levels of brain activation (Figure 5) and only local brain activation regions (Figure 8a; channel 3, 4, 8, and 9) were detected.

Region BA46 in the left DLPFC is associated with attention function [28,29]. According to Grundy’s meta-analysis, bilingualism is related to working memory [30]. The prefrontal cortex is also involved in executive function of higher-order functions [31]. Among them, the execution function consists of the inhibition function, code-switching function, and information update [32]. This suggests that L2 learning can change the cerebral blood flow dynamics of the prefrontal cortex.

Activation of the left hemisphere in all subjects in this study is consistent with the involvement of left DLPFC (BA46) in language production [29]. Forstmann and colleagues conducted an experiment using the Simon test that required inhibition of responses to incongruent stimuli, they found that those who were proficient in inhibiting responses showed increased structural connectivity in the right inferior frontal gyrus (IFG), reflecting higher density of white matter [33]. The present study revealed activation of the right DLPFC, suggesting L1 inhibition, which is consistent with the results of a study by Van Ettinger et al., and findings that performance in high-level language tests was related to increased activity in the IFG [34]. Moreover, the results of the present work are compatible with those of a code-switching task in bilingual speakers of Korean and Chinese, during which activation of the left frontal cortex and upper right frontal cortex was confirmed [35].

The present study confirmed that brain blood flow was changed by language learning, especially that involved in inhibition of L1 and L2 in high-L2-ability speakers. Behavior inhibition was demonstrated to be associated with the right DLPFC, and language learning was associated with the right frontal cortex, which is considered to be involved in language learning and behavior inhibition [33,36]. Brain activation was markedly revealed at both the right DLPFC and the left DLPFC in high-L2-ability speakers in this study, strongly suggesting the involvement of right DLPFC with L2 language proficiency.

### 4.4. Mutual Influence of Language Distance

Language distance in the brain is a factor affecting L2 learning. In general, L2 learning is easier when the L1/L2 language distance is close [37]. However, some studies also found that close language distance causes mutual interference in code-switching and inhibition [35,38]. Since the language distance between Japanese and Mandarin is close, they mutually affect each other. To draw out such an influence, the same *Kanji* was used to confirm brain activity.

Mandarin and Japanese bilinguals simultaneously activate two similar language systems, and two processing departments in lemma level and lexeme level, occurring in two directions. Bilinguals demonstrate greater cognitive load in inhibition and code-switching to select the right language to respond to complex information in language processing [39,40]. Furthermore, to inhibit unwanted behavior, the dorsolateral prefrontal cortex (DLPFC BA9 and BA46: ch5, 9, 10, and 13) is involved in selecting the appropriate behavior [34].

In the present study, the same *Kanji* was used in both the Mandarin and Japanese tasks; therefore, the dominant language should easily appear. In particular, the high-L2-ability speakers preferred to use Japanese in the Mandarin task. It has been reported that brain activation related to inhibition of behavior occurs in the right lateral prefrontal cortex [36]. Sometimes during performance of the L1 task, high-L2-ability speakers used Japanese subconsciously, it would seem that they prefer it to their mother-tongue Mandarin. Therefore, inhibition was required for Japanese, and the right frontal cortex was activated more. Involvement of the prefrontal cortex in language learning affects cognitive control [41,42], and higher levels of metacognition [43,44] than cognitive reserve [45,46].

### 4.5. Study Limitations and Prospects

This study had some limitations, the number of subjects was small, only 12 in each L2 ability group, which we selected to ensure within-group similarity in cognitive reserve. There was a significant age difference between groups, which was necessary to discriminate between high and low L2 ability developed after reaching adulthood. Additionally, areas of the brain beyond the prefrontal cortex were not measured.

On the other hand, all subjects lived and functioned in a bilingual environment in Japan with highly unified social and economic factors, which suggests high reliability of the study findings. The strong correlations between L2 and cognitive function suggest learning a second language would be helpful to significantly delay the onset of dementia by changing brain activation pattern [2,15,47,48].

## 5. Conclusions

A novel Mandarin (L1) Japanese (L2) speaking task system was developed and applied to evaluate brain activation during performance of a speaking task by people who can speak both Mandarin and Japanese. Cerebral blood flow change was revealed in the prefrontal cortex by measuring oxygen levels using fNIRS. The relationship between prefrontal cortex blood flow change and L2 proficiency was discussed. The results obtained were as follows:

1. People with low L2 ability showed much more brain activation when speaking L2 than when speaking L1. People with high L2 ability showed high-level brain activation when speaking either L2 or L1. Almost the same high-level brain activation was observed in both ability groups when speaking L2.
2. The high level of activation in people with high L2 ability when speaking either L2 or L1 suggested strong inhibition of the non-spoken language. A wider area of brain activation in people with low compared with high L2 ability when speaking L2 is considered to be attributed to the cognitive load involved in code-switching L1 to L2 with strong inhibition of L1 and the cognitive load involved in using L2.
3. The above results suggest that learning a second language of Japanese would be helpful for Chinese speakers of Mandarin to delay the onset of dementia by changing brain activation pattern. This effect should also be furtherly confirmed through an analysis of a wider area of the brain of more subjects using the fNIRS measurement as well as other techniques. Furthermore, implications for the fields of neurolinguistics and language education are also expected. An effective method for language education in enhancing the cognitive function might be important.

## Figures and Tables

**Table 1:** Characteristics of study participants.

All Subjects Are Native Speakers of Mandarin (L1) with Japanese as a Second Language (L2)				
Characteristics		Group 1 (n = 12)	Group 2 (n = 12)	*p*-Value
Age (years: mean ± SD)		51.1 ± 5.0	24.9 ± 1.4	<0.0001
Sex (female/male)		5/7	6/6	
Living years in Japan (years: mean ± SD)		22.3 ± 3.5	2.75 ± 1.1	<0.0001
AOA * (years: mean ± SD)		27.3 ± 2.5	22.4 ± 0.5	<0.0001
Japanese (L2)	Reading (mean score ± SD)	9.2 ± 0.4	4.2 ± 1.8	<0.0001
	Listening (mean score ± SD)	9.2 ± 0.7	4.0 ± 1.8	<0.0001
	Writing (mean score ± SD)	8.1 ± 1.2	2.8 ± 1.8	<0.0001
	Speaking (mean score ± SD)	8.5 ± 1.2	3.4 ± 1.8	<0.0001
	total-Japanese (mean score ± SD)	8.8 ± 0.7	3.6 ± 1.7	<0.0001
Mandarin (L1)	Reading (mean score ± SD)	9.4 ± 0.9	9.3 ± 0.9	=0.8215
	Listening (mean score ± SD)	9.5 ± 0.7	9.8 ± 0.4	=0.150
	Writing (mean score ± SD)	9.0 ± 0.6	9.0 ± 1.1	=1.0000
	Speaking (mean score ± SD)	9.5 ± 0.5	9.7 ± 0.5	=0.3140
	total-Japanese (mean score ± SD)	9.4 ± 0.5	9.5 ± 0.6	=0.6553
Criq **	CRiq-E *** (mean score ± SD)	132 ± 2	102 ± 5	<0.0001
	CRiq-W **** (mean score ± SD)	108 ± 15	91 ± 1	=0.0006
	CRiq-L ***** (mean score ± SD)	107 ± 8	89 ± 1	<0.0001
	total-CRiq (mean score ± SD)	120 ± 8	92 ± 3	<0.0001
group 1 = High L2 ability group 2 = Low L2 ability				

* AOA = age of acquisition of L2. ** CRiq = Cognitive Reserve Index questionnaire; *** E = Education; **** W = Working Activity; ***** L = Leisure Time [19]. Scores: L1 and L2 scores from self-assessment questionnaire previously described [16,17,18].

**Table 2:** Values of Δoxy-Hb at 22 channels in high- and low-L2-ability speakers when speaking Mandarin (L1) or Japanese (L2): red indicates significant between-group differences (*p* < 0.05).

	L1 Task					L2 Task				
	High-L2-Ability Group		Low-L2-Ability Group			High-L2-Ability Group		Low-L2-Ability Group		
Channel	Mean	SD	Mean	SD	*p*-Value	Mean	SD	Mean	SD	*p*-Value
ch1	0.739	0.621	−0.0570	0.705	0.0077	0.641	0.863	0.837	0.855	0.5819
ch2	0.606	0.636	−0.1490	0.660	0.0093	0.532	0.946	0.414	0.843	0.7508
ch3	0.611	0.619	0.1130	0.569	0.0517	0.585	0.806	0.537	0.879	0.8892
ch4	0.840	1.065	0.1810	0.719	0.0894	0.748	1.370	0.936	1.150	0.719
ch5	0.876	0.659	−0.2160	0.818	0.0016	0.699	0.833	0.698	1.180	0.9984
ch6	0.516	0.653	−0.4860	0.772	0.0024	0.318	0.100	0.350	1.004	0.9376
ch7	0.487	0.507	−0.0930	0.581	0.0162	0.327	0.801	0.460	0.775	0.6841
ch8	0.663	0.725	0.2160	0.649	0.1258	0.508	1.081	0.827	0.820	0.4248
ch9	1.094	0.885	0.4080	0.588	0.0358	0.729	1.246	1.252	0.976	0.265
ch10	0.621	0.664	−0.5850	0.732	0.0003	0.364	0.653	0.442	1.192	0.8451
ch11	0.618	0.459	−0.2250	0.533	0.0004	0.422	0.598	0.680	0.936	0.4287
ch12	0.672	0.520	0.0580	0.529	0.009	0.630	0.664	0.692	0.725	0.829
ch13	1.132	0.674	0.3490	0.685	0.0099	0.894	0.290	1.420	0.290	0.2126
ch14	0.851	0.804	0.0500	0.427	0.0059	0.528	0.587	0.745	1.267	0.5944
ch15	0.769	0.927	−0.4180	0.492	0.0007	0.539	1.007	0.992	1.262	0.3415
ch16	1.351	1.081	−0.1440	0.984	0.0018	1.102	1.105	1.267	1.064	0.7129
ch17	1.070	0.853	−0.0160	0.480	0.0009	0.845	0.953	1.073	1.059	0.5858
ch18	1.005	0.703	0.2550	0.575	0.0091	0.956	0.775	1.158	1.002	0.5856
ch19	1.135	0.867	−0.1270	0.909	0.0021	1.056	1.146	0.749	0.841	0.4626
ch20	1.200	1.074	−0.2870	1.024	0.0022	1.130	1.094	0.693	1.027	0.3242
ch21	1.201	1.199	−0.2430	0.869	0.0027	1.221	1.044	0.784	1.079	0.3244
ch22	0.951	0.757	0.0020	0.711	0.0044	1.119	0.980	0.712	1.038	0.3339
Total right	7.932	6.379	−2.4990	4.791	0.0002	6.229	7.453	6.602	8.604	0.9106
Total left	9.239	6.990	1.3200	4.209	0.0028	8.236	8.480	9.391	7.542	0.7277

**Table 3:** Values of Δoxy-Hb at 22 channels in high-L2-ability speakers when speaking Mandarin (L1) or Japanese (L2).

L1 Task			L2 Task		
Channel	Mean	SD	Mean	SD	*p*-Value
ch1	0.739	0.621	0.641	0.863	0.7526
ch2	0.606	0.636	0.532	0.946	0.8248
ch3	0.611	0.619	0.585	0.806	0.9310
ch4	0.840	1.065	0.748	1.370	0.8555
ch5	0.876	0.659	0.699	0.833	0.5693
ch6	0.516	0.653	0.318	0.100	0.5706
ch7	0.487	0.507	0.327	0.801	0.5664
ch8	0.663	0.725	0.508	1.081	0.6850
ch9	1.094	0.885	0.729	1.246	0.4180
ch10	0.621	0.664	0.364	0.653	0.3492
ch11	0.618	0.459	0.422	0.598	0.3780
ch12	0.672	0.520	0.630	0.664	0.8660
ch13	1.132	0.674	0.894	0.290	0.4874
ch14	0.851	0.804	0.528	0.587	0.2721
ch15	0.769	0.927	0.539	1.007	0.5659
ch16	1.351	1.081	1.102	1.105	0.5832
ch17	1.07	0.853	0.845	0.953	0.5486
ch18	1.005	0.703	0.956	0.775	0.8723
ch19	1.135	0.867	1.056	1.146	0.8506
ch20	1.200	1.074	1.130	1.094	0.8769
ch21	1.201	1.199	1.221	1.044	0.9648
ch22	0.951	0.757	1.119	0.98	0.6439
Total right	7.932	6.379	6.229	7.453	0.5539
Total left	9.239	6.990	8.236	8.480	0.7549

**Table 4:** Values of Δoxy-Hb at 22 channels in low-L2-ability speakers when speaking Mandarin (L1) or Japanese (L2). Red indicates significant between-task differences (*p* < 0.05).

L1 Task			L2 Task		
Channel	Mean	SD	Mean	SD	*p*-Value
ch1	−0.057	0.705	0.837	0.855	0.0106
ch2	−0.149	0.660	0.414	0.843	0.0821
ch3	0.113	0.569	0.537	0.879	0.1742
ch4	0.181	0.719	0.936	1.150	0.0667
ch5	−0.216	0.818	0.698	1.180	0.0381
ch6	−0.486	0.772	0.350	1.004	0.0322
ch7	−0.093	0.581	0.460	0.775	0.0605
ch8	0.216	0.649	0.827	0.820	0.0552
ch9	0.408	0.588	1.252	0.976	0.0176
ch10	−0.585	0.732	0.442	1.192	0.0185
ch11	−0.225	0.533	0.680	0.936	0.0081
ch12	0.058	0.529	0.692	0.725	0.0227
ch13	0.349	0.685	1.420	0.290	0.0073
ch14	0.050	0.427	0.745	1.267	0.0855
ch15	−0.418	0.492	0.992	1.262	0.0016
ch16	−0.144	0.984	1.267	1.064	0.0027
ch17	−0.016	0.480	1.073	1.059	0.0037
ch18	0.255	0.575	1.158	1.002	0.0013
ch19	−0.127	0.909	0.749	0.841	0.0266
ch20	−0.287	1.024	0.693	1.027	0.0287
ch21	−0.243	0.869	0.784	1.079	0.0175
ch22	0.002	0.711	0.712	1.038	0.0630
Total right	−2.499	4.791	6.602	8.604	0.0041
Total left	1.320	4.209	9.391	7.542	0.0038

## Data Availability

The data presented in this study are available on request from the corresponding author. The data are not publicly available due to ethical guideline.

## References

[1] Sakai K.L. (2005). Language acquisition and brain development. Science.

[2] Antoniou M., Gunasekera G.M., Wong P.C. (2013). Foreign language training as cognitive therapy for age-related cognitive decline: A hypothesis for future research. Neurosci. Biobehav. Rev..

[3] Rodriguez-Fornells A., Cunillera T., Mestres-Misse A., de Diego-Balaguer R. (2009). Neurophysiological mechanisms involved in language learning in adults. Philos. Trans. R. Soc. Lond. B Biol. Sci..

[4] Hosoda C., Tanaka K., Nariai T., Honda M., Hanakawa T. (2013). Dynamic neural network reorganization associated with second language vocabulary acquisition: A multimodal imaging study. J. Neurosci..

[5] Legault J., Fang S.Y., Lan Y.J., Li P. (2018). Structural brain changes as a function of second language vocabulary training: Effects of learning context. Brain Cogn..

[6] Del Maschio N., Sulpizio S., Gallo F., Fedeli D., Weekes B.S., Abutalebi J. (2018). Neuroplasticity across the lifespan and aging effects in bilinguals and monolinguals. Brain Cogn..

[7] Li P., Legault J., Litcofsky K.A. (2014). Neuroplasticity as a function of second language learning: Anatomical changes in the human brain. Cortex.

[8] Alladi S., Bak T.H., Duggirala V., Surampudi B., Shailaja M., Shukla A.K., Chaudhuri J.R., Kaul S. (2013). Bilingualism delays age at onset of dementia, independent of education and immigration status. Neurology.

[9] Rose N.S., Luo L., Bialystok E., Hering A., Lau K., Craik F.I. (2015). Cognitive processes in the Breakfast Task: Planning and monitoring. Can. J. Exp. Psychol..

[10] Schweizer T.A., Ware J., Fischer C.E., Craik F.I., Bialystok E. (2012). Bilingualism as a contributor to cognitive reserve: Evidence from brain atrophy in Alzheimer’s disease. Cortex.

[11] Ardila A., Bernal B., Rosselli M. (2016). Connectivity of BA46 involvement in the executive control of language. Psicothema.

[12] De Baene W., Duyck W., Brass M., Carreiras M. (2015). Brain Circuit for Cognitive Control is Shared by Task and Language Switching. J. Cogn. Neurosci..

[13] Craik F.I.M. (2017). Cognitive Problems in Older Adults: Can Bilingualism Help? Chapter 2.

[14] Green D.W., Abutalebi J. (2013). Language control in bilinguals: The adaptive control hypothesis. J. Cogn. Psychol..

[15] Nickels L., Hameau S., Nair V.K.K., Barr P., Biedermann B. (2019). Ageing with bilingualism: Benefits and challenges. Speech Lang. Hear..

[16] Li P., Sepanski S., Zhao X. (2006). Language history questionnaire: A web-based interface for bilingual research. Behav. Res. Methods.

[17] Calabria M., Branzi F.M., Marne P., HernÁNdez M., Costa A. (2013). Age-related effects over bilingual language control and executive control. Biling. Lang. Cogn..

[18] Borragan M., Martin C.D., de Bruin A., Dunabeitia J.A. (2018). Exploring Different Types of Inhibition During Bilingual Language Production. Front. Psychol..

[19] Nucci M., Mapelli D., Mondini S. (2012). Cognitive Reserve Index questionnaire (CRIq): A new instrument for measuring cognitive reserve. Aging Clin. Exp. Res..

[20] Endo K., Liang N., Idesako M., Ishii K., Matsukawa K. (2018). Incremental rate of prefrontal oxygenation determines performance speed during cognitive Stroop test: The effect of ageing. J. Physiol. Sci..

[21] Tamashiro H., Kinoshita S., Okamoto T., Urushidani N., Abo M. (2018). Effect of baseline brain activity on response to low-frequency rTMS/intensive occupational therapy in poststroke patients with upper limb hemiparesis: A near-infrared spectroscopy study. Int. J. Neurosci..

[22] Kondo A., Shoji Y., Morita K., Sato M., Ishii Y., Yanagimoto H., Nakano S., Uchimura N. (2018). Characteristics of oxygenated hemoglobin concentration change during pleasant and unpleasant image-recall tasks in patients with depression: Comparison with healthy subjects. Psychiatry Clin. Neurosci..

[23] Ye J.C., Tak S., Jang K.E., Jung J., Jang J. (2011). NIRS-SPM: Statistical Parametric Mapping for Near-infrared Spectroscopy. Neuroimage.

[24] García A.M., Muñoz E., Kogan B. (2019). Taxing the bilingual mind: Effects of simultaneous interpreting experience on verbal and executive mechanisms. Biling. Lang. Cogn..

[25] Klein D., Mok K., Chen J.K., Watkins K.E. (2014). Age of language learning shapes brain structure: A cortical thickness study of bilingual and monolingual individuals. Brain Lang..

[26] Mohades S.G., Struys E., Van Schuerbeek P., Mondt K., Van De Craen P., Luypaert R. (2012). DTI reveals structural differences in white matter tracts between bilingual and monolingual children. Brain Res..

[27] Kakimoto Y., Nishimura Y., Hara N., Okada M., Tanii H., Okazaki Y. (2009). Intrasubject reproducibility of prefrontal cortex activities during a verbal fluency task over two repeated sessions using multi-channel near-infrared spectroscopy. Psychiatry Clin. Neurosci..

[28] MacDonald A.W., Cohen J.D., Stenger V.A., Carter C.S. (2000). Dissociating the Role of the Dorsolateral Prefrontal and Anterior Cingulate Cortex in Cognitive Control. Science.

[29] Klaus J., Schutter D. (2018). The Role of Left Dorsolateral Prefrontal Cortex in Language Processing. Neuroscience.

[30] Grundy J.G., Timmer K. (2016). Bilingualism and working memory capacity: A comprehensive meta-analysis. Second Lang. Res..

[31] Alvarez J.A., Emory E. (2006). Executive function and the frontal lobes: A meta-analytic review. Neuropsychol. Rev..

[32] Godefroy O., Jeannerod M., Allain P., Le Gall D. (2018). Lobe frontal, fonctions exécutives et controle cognitif Frontal lobe, executive functions and cognitive contro. Rev. Neuroloque.

[33] Forstmann B.U., Jahfari S., Scholte H.S., Wolfensteller U., van den Wildenberg W.P., Ridderinkhof K.R. (2008). Function and structure of the right inferior frontal cortex predict individual differences in response inhibition: A model-based approach. J. Neurosci..

[34] Van Ettinger-Veenstra H., Ragnehed M., McAllister A., Lundberg P., Engstrom M. (2012). Right-hemispheric cortical contributions to language ability in healthy adults. Brain Lang..

[35] Lei M., Akama H., Murphy B. (2014). Neural basis of language switching in the brain: fMRI evidence from Korean-Chinese early bilinguals. Brain Lang..

[36] Kinoshita S., Tamashiro H., Okamoto T., Urushidani N., Abo M. (2019). Association between imbalance of cortical brain activity and successful motor recovery in sub-acute stroke patients with upper limb hemiparesis: A functional near-infrared spectroscopy study. Neuroreport.

[37] Chiswick B.R., Miller P.W. (2005). Linguistic Distance: A Quantitative Measure of the Distance between English and Other Languages. J. Multiling. Multicult. Dev..

[38] Ghazi-Saidi L., Ansaldo A.I. (2017). Second Language Word Learning through Repetition and Imitation: Functional Networks as a Function of Learning Phase and Language Distance. Front. Hum. Neurosci..

[39] Costa A., Santesteban M. (2004). Lexical access in bilingual speech production: Evidence from language switching in highly proficient bilinguals and L2 learners. J. Mem. Lang..

[40] Linck J.A., Kroll J.F., Sunderman G. (2009). Losing access to the native language while immersed in a second language: Evidence for the role of inhibition in second-language learning. Psychol. Sci..

[41] Sullivan M.D., Janus M., Moreno S., Astheimer L., Bialystok E. (2014). Early stage second-language learning improves executive control: Evidence from ERP. Brain Lang..

[42] Jasinska K.K., Petitto L.A. (2014). Development of neural systems for reading in the monolingual and bilingual brain: New insights from functional near infrared spectroscopy neuroimaging. Dev. Neuropsychol..

[43] Peter Bright R.F. (2019). Perspectives on the ‘Bilingual Advantage’: Challenges and Opportunities. Front. Psychol..

[44] Dash T., Ghazi-Saidi L., Berroir P., Adrover-Roig D., Benali H., Ansaldo A.I. (2017). Is the bilingual brain better equipped for aging. OLBI J..

[45] Stern Y. (2002). What is cognitive reserve? Theory and research application of the reserve concept. J. Int. Neuropsychol. Soc..

[46] Stern Y. (2009). Cognitive reserve. Neuropsychologia.

[47] Craik F.I., Bialystok E., Freedman M. (2010). Delaying the onset of Alzheimer disease: Bilingualism as a form of cognitive reserve. Neurology.

[48] Guzman-Velez E., Tranel D. (2015). Does bilingualism contribute to cognitive reserve? Cognitive and neural perspectives. Neuropsychology.

